# Associations Between Polymorphisms in the Glucocorticoid-Receptor Gene and Cardiovascular Risk Factors in a Chinese Population

**DOI:** 10.2188/jea.JE20130035

**Published:** 2013-09-05

**Authors:** Yu-Xiang Yan, Jing Dong, Li-Juan Wu, Shuang Shao, Jie Zhang, Ling Zhang, Wei Wang, Yan He, You-Qin Liu

**Affiliations:** 1 Department of Epidemiology and Biostatistics, School of Public Health, Capital Medical University, Beijing, China; 2 Municipal Key Laboratory of Clinical Epidemiology, Beijing, China; 3 Physical Examination Center, Beijing Xuanwu Hospital, Capital Medical University, Beijing, China

**Keywords:** glucocorticoid receptor gene, polymorphism, cardiovascular disease, risk factor

## Abstract

**Background:**

Glucocorticoid is an important regulator of energy homeostasis. Glucocorticoid receptor (GR) gene polymorphisms that contribute to variability in glucocorticoid sensitivity have been identified. We explored the associations of single-nucleotide polymorphisms (SNPs) of the GR gene with traditional cardiovascular risk factors in the Chinese Han population.

**Methods:**

We recruited 762 consecutive adults who underwent a regular physical examination at Beijing Xuanwu Hospital. Blood pressure, glucose, lipid levels (total cholesterol, high-density lipoprotein cholesterol, low-density lipoprotein [LDL] cholesterol and triglycerides), body mass index (BMI), and waist-to-hip ratio were measured. Fourteen tag SNPs and 5 functional SNPs were selected and genotyped using the high-throughput Sequenom genotyping platform. Differences between genotypes/alleles for each SNP were adjusted for sex and age and tested using a general linear model procedure. Various models of inheritance, including additive, dominant, and recessive, were tested.

**Results:**

Among the 19 SNPs examined, 5 markers were associated with cardiovascular risk factors. The rs41423247 GG genotype and the rs7701443 AA genotype were associated with higher BMI and systolic blood pressure (*P* < 0.0004), and the rs17209251 GG genotype was associated with higher systolic blood pressure (*P* < 0.0004). Lower systolic blood pressure, total cholesterol, and LDL cholesterol were observed among rs10052957 A allele carriers (*P* < 0.0004), and lower plasma glucose and LDL-cholesterol concentrations were observed among rs2963156 TT carriers (*P* < 0.0004).

**Conclusions:**

Polymorphism of the GR gene was associated with cardiovascular risk factors and may contribute to susceptibility to cardiovascular disease.

## INTRODUCTION

The glucocorticoids (GCs) are important regulators of energy homeostasis. Most effects of GCs are mediated by the glucocorticoid receptor (GR),^1^ which consists of a hormone-binding domain, a DNA-binding domain, and the amino-terminal region^2^ and has a role in determining GC sensitivity. Alterations in any of the above molecular mechanisms of GR action can change tissue sensitivity to GC, possibly resulting in resistance or hypersensitivity and substantial morbidity.^3^^–^^5^

China has experienced an epidemic of cardiovascular disease in recent decades, and available evidence suggests that genetic mechanisms contribute to the incidence of such disease. Several polymorphisms of the GR gene (NR3C1) have been reported to be associated with cardiovascular disease.^1^^,^^6^ The N363S polymorphism results from an amino acid substitution from asparagine (N) to serine (S) at codon 363 and is associated with increased sensitivity to GC in vivo^6^^–^^8^ and higher body mass index (BMI)^6^^,^^9^^,^^10^ and waist-to-hip ratio.^11^ The N363S variant is also associated with elevated cholesterol and triglyceride concentrations and higher incidence of coronary artery disease, independent of weight.^12^^,^^13^ The *Bcl* I polymorphism is a relatively common variant and has been reported to be associated with increased sensitivity to GC, hypertension,^6^^,^^14^^,^^15^ and increased insulin concentrations among obese women.^16^ The ER22/23EK polymorphism consists of 2 linked point mutations, at codons 22 and 23: a change from GAG to GAA at codon 22, which result in both coding for glutamic acid (E), and a change from AGG to AAG at codon 23, which results in an amino acid substitution from arginine (R) to lysine (K).^17^ This polymorphism is associated with GC resistance, lower fasting insulin concentrations, and lower concentrations of total cholesterol, low-density lipoprotein (LDL) cholesterol, and C-reactive protein (CRP).^17^^–^^19^ GC resistance and the healthier metabolic profiles observed among Tth111I carriers are likely due to the ER22/23EK polymorphism.^20^ A novel point mutation of receptor GRα (D401H) enhances transcriptional activity of GR-mediated genes. Presence of the D401H mutation may predispose individuals to an adverse metabolic profile and increase the risk of metabolic syndrome-related atherosclerotic cardiovascular disease.^21^ The GRβ polymorphism A3669G, in the 3′ untranslated region, results in enhanced expression of the dominant-negative GRβ and is associated with favorable metabolic parameters.^22^ However, the relationship between the GR gene and cardiovascular disease varies substantially among populations because the frequency and effects of polymorphisms differ greatly by race.^6^

To our knowledge, few studies have investigated GR gene polymorphisms in the Chinese population. To clarify the associations of these polymorphisms with cardiovascular risk factors, we comprehensively examined variation across the GR gene in the Chinese Han population.

## METHODS

### Study participants

From January to May 2012, we conducted a cross-sectional study at the physical examination center of Xuanwu Hospital, Capital Medical University. Individuals who underwent regular physical examinations for at least 2 consecutive years were recruited consecutively. The study inclusion criteria were as follows: (1) no history of somatic or psychiatric abnormalities, as confirmed by patient medical records, (2) age 20 through 60 years, and (3) no history of medication use during the previous 2 weeks. Patients were excluded if they had previously received a diagnosis of specific diseases of the cardiovascular, respiratory, genitourinary, digestive, or hematologic system. Ultimately, 762 Han Chinese residing in Beijing were enrolled. To ensure comparability of the findings, all participants were examined by physicians who were specially trained for the study. All participants signed an informed consent form, and this study was approved by the Ethical Committee of Capital Medical University.

### Data collection

Classical risk factors, ie, blood pressure, glucose, lipid levels, BMI, and waist-to-hip ratio (WHR), were included in the analyses. Height, weight, waist circumference, and hip circumference were also measured. BMI was calculated as weight in kilograms divided by the square of the height in meters (kg/m^2^). WHR was calculated by dividing waist circumference by hip circumference. While participants were seated, a standard mercury sphygmomanometer was used to measure blood pressure 3 times consecutively after 5 minutes of rest. The average of the second and third measurements was used in the analyses. For laboratory measurements and genotype analysis, fasting blood samples were collected by venipuncture in the morning after an overnight fast of at least 12 hours. Concentrations of total and high-density lipoprotein (HDL) cholesterol and triglycerides were measured using standard laboratory methods (Hitachi automatic clinical analyzer, model 7060, Japan); LDL cholesterol levels were calculated using the Friedewald formula. Fasting plasma glucose (FPG) levels were measured by the glucose oxidase method.

### Selection of single-nucleotide polymorphisms and genotyping

Tag single-nucleotide polymorphisms (SNPs) were selected between 5000 bp upstream of the initiation codon and 5000 bp downstream of the termination codon of NR3C1, using genotype data from the CHB (Han Chinese in Beijing) panel of the Phase II HapMap database. The criteria for tagging were a linkage disequilibrium (LD) threshold (r^2^) greater than or equal to 0.8 and a minor allele frequency (MAF) greater than or equal to 0.1. In addition to the 14 tag SNPs (Table 2), 5 other SNPs (N363S, *Bcl* I, ER22/23EK, A3669G, and D401H) were added to the set of markers, based on their reported functional/clinical significance.^12^^–^^19^^,^^21^^,^^22^ These polymorphisms have been shown to be associated with cardiovascular disease.

**Table 2.** Associations of cardiovascular risk factors with rs41423247 genotypes/alleles

Variable	CC (*n* = 486)	CG (*n* = 246)	GG (*n* = 30)	*P* (Additive model)	*P* (Dominant model^a^)	*P* (Recessive model^b^)
Age (years)	41.80 ± 10.54	41.96 ± 10.83	40.70 ± 11.13			
BMI	23.59 ± 3.13	23.20 ± 2.66	25.50 ± 2.54	3.1 × 10^−4^	0.557	1.6 × 10^−4^
WHR	0.81 ± 0.06	0.81 ± 0.05	0.82 ± 0.06	0.815	0.939	0.528
SBP (mm Hg)	112.57 ± 11.62	113.84 ± 12.73	122.90 ± 12.57	2.7 × 10^−5^	0.017	1.1 × 10^−5^
DBP (mm Hg)	74.31 ± 8.17	74.92 ± 8.62	79.30 ± 6.70	0.005	0.080	0.001
GLU (mmol/L)	5.38 ± 0.77	5.30 ± 0.57	5.06 ± 0.38	0.020	0.017	3.0 × 10^−4^
TC (mmol/L)	4.74 ± 0.87	4.78 ± 0.85	4.97 ± 0.83	0.326	0.337	0.172
TG (mmol/L)	1.23 ± 0.89	1.19 ± 0.92	0.96 ± 0.22	0.229	0.308	2.7 × 10^−4^
HDL-C (mmol/L)	1.64 ± 0.33	1.62 ± 0.30	1.69 ± 0.19	0.503	0.797	0.094
LDL-C (mmol/L)	2.56 ± 0.70	2.65 ± 0.69	2.62 ± 0.71	0.254	0.099	0.772

BMI: body mass index, WHR: waist-to-hip ratio, SBP: systolic blood pressure, DBP: diastolic blood pressure, GLU: plasma glucose, TC: total cholesterol, TG: triglyceride, HDLC: high-density lipoprotein cholesterol, LDLC: low-density lipoprotein cholesterol.

^a^Dominant model: CG+GG vs CC.

^b^Recessive model: GG vs CC+CG.

Genomic DNA was extracted from peripheral white blood cells of participants, using a DNeasy tissue kit (Qiagen), according to the manufacturer’s instructions, and the 19 SNPs were genotyped using the high-throughput Sequenom genotyping platform. Personnel performing genotyping were unaware of the phenotype of interest. Genotyping was repeated in 5% of samples, for verification and quality control. Quality control testing showed that the genotype data had an error rate of less than 0.1%.

### Statistical analyses

Genotype and allele frequencies were determined with the gene counting method. The χ^2^ test was used to calculate the Hardy-Weinberg equilibrium (HWE) and distributions of categorical variables. The clinical characteristics of participants were analyzed as continuous variables. Differences between genotypes/alleles for each SNP were adjusted for sex and age and tested using the general linear model procedure. The least-significant difference (LSD) *t*-test was used to adjust for multiple comparisons. Various models of inheritance (additive, dominant, and recessive) were tested. For dominant effects, test-allele carriers were compared with noncarriers; for recessive effects, participants homozygous for the test allele were compared with heterozygous carriers and noncarriers. All statistical analysis was done using SPSS 13.0 (SPSS Inc., Chicago, IL, USA). *P* values were corrected using the Bonferroni method for single-marker analyses.

## RESULTS

The mean (SD) age of the 762 participants was 41.81 (10.64) years (range 22–60 years). There were 300 men (39.4%) and 462 women (60.6%). The overall genotyping rate for the 19 SNPs was 99.7%. Regarding the 5 functional SNPs, no carriers of N363S, ER22/23EK, A3669G, and D401H were found in the sample. The final analysis was thus based on 15 SNPs. The multiple test-corrected threshold for α was 0.0004, assuming a family-wise α of 0.05, 15 markers, and 9 cardiovascular risk factors. All 14 tag SNPs (Table 1) were in Hardy-Weinberg equilibrium. Among the 14 tag SNPs, 4 were significantly associated with cardiovascular risk factors. No significant associations with the other 10 tag SNPs were observed.

**Table 1.** Characteristics of investigated tag SNPs in the GR gene

SNP ID	Genomic location	Gene position	Alleles (major/minor)	MAF	HWE *P*
rs10052957	142766894	intron_1	G/A	0.122	0.268
rs17100289	142788835	intron_1	A/T	0.478	0.326
rs7701443	142772843	intron_1	G/A	0.400	0.802
rs12054797	142786095	intron_1	C/T	0.100	0.841
rs6865292	142773183	intron_1	T/C	0.288	0.893
rs9324924	142772677	intron_1	G/T	0.389	0.719
rs4607376	142776725	intron_1	G/A	0.389	0.991
rs6877893	142707386	intron_2	A/G	0.278	0.989
rs17100236	142700919	intron_2	T/C	0.170	0.932
rs2963155	142736197	intron_2	A/G	0.233	0.411
rs2963156	142738689	intron_2	C/T	0.156	0.171
rs4912905	142710569	intron_2	C/G	0.341	0.381
rs9324921	142747933	intron_2	C/A	0.233	0.081
rs17209251	142649416	intron_7	A/G	0.178	0.454

MAF: minor allele frequency, HWE: Hardy-Weinberg equilibrium.

Our data showed that the allele frequency of the SNP rs41423247 (*Bcl* I) polymorphism was 79.9% for the C allele and 20.1% for the G allele, which was in agreement with the Hardy-Weinberg equilibrium (*P* = 0.991). Table 2 shows the mean (SD) values for cardiovascular risk factors among the study groups, according to rs41423247 genotype. Under the additive model, BMI and systolic blood pressure (SBP) significantly differed among the 3 genotypes, after adjustment for age and sex (*P* < 0.0004). After adjustment for multiple comparisons, the differences between the CG and CC genotypes were not statistically significant (*P* > 0.0004). The dominant model showed no significant difference between G allele carriers and noncarriers for any tested trait (*P* > 0.0004). The recessive model showed that the GG genotype was associated with higher BMI and SBP, and lower plasma glucose and triglycerides, as compared with C allele carriers (*P* < 0.0004).

For SNP rs10052957 (Tth111I), the genotype frequencies for GG, GA, and AA were 605 (79.4%), 153 (20.1%), and 4 (0.5%), respectively. Only the dominant model was used to compare the parameters (Table 3). As compared with the GG genotype, A allele carriers had significantly lower SBP, total cholesterol, and LDL cholesterol (*P* < 0.0004).

**Table 3.** Associations of cardiovascular risk factors with rs10052957 GG and GA/AA genotypes

Variable	GG (*n* = 605)	GA+AA (*n* = 157)	*P* (Dominant model^a^)
Age (years)	43.83 ± 10.70	41.23 ± 10.57	
BMI	23.68 ± 2.53	23.51 ± 3.11	0.470
WHR	0.81 ± 0.05	0.81 ± 0.06	0.536
SBP (mm Hg)	116.74 ± 11.83	112.62 ± 12.12	2.1 × 10^−4^
DBP (mm Hg)	75.79 ± 7.93	74.45 ± 8.40	0.080
GLU (mmol/L)	5.34 ± 0.63	5.41 ± 0.99	0.816
TC (mmol/L)	5.05 ± 0.88	4.68 ± 0.84	1.9 × 10^−6^
TG (mmol/L)	1.28 ± 0.96	1.22 ± 1.05	0.401
HDL-C (mmol/L)	1.67 ± 0.30	1.62 ± 0.32	0.110
LDL-C (mmol/L)	2.80 ± 0.70	2.53 ± 0.69	1.8 × 10^−5^

BMI: body mass index, WHR: waist-to-hip ratio, SBP: systolic blood pressure, DBP: diastolic blood pressure, GLU: plasma glucose, TC: total cholesterol, TG: triglyceride, HDLC: high-density lipoprotein cholesterol, LDLC: low-density lipoprotein cholesterol.

^a^Dominant model: GA+AA vs GG.

The SNP rs7701443 was significantly associated with BMI and SBP (Table 4). As compared with the GG and GA genotypes, the AA genotype was significantly associated with higher BMI and SBP (*P* < 0.0004); however, there was no significant difference between the GA and AA genotypes (*P* > 0.0004). In the recessive model, AA homozygotes had a significantly higher BMI and SBP than did carriers of G alleles (*P* < 0.0004). In the dominant model, there was no significant difference between carriers and noncarriers of the A allele (*P* > 0.0004).

**Table 4.** Associations of cardiovascular risk factors with rs7701443 genotypes/alleles

Variable	GG (*n* = 301)	GA (*n* = 365)	AA (*n* = 96)	*P* (Additive model)	*P* (Dominant model^a^)	*P* (Recessive model^b^)
Age (years)	40.80 ± 10.34	42.22 ± 10.93	43.40 ± 10.28			
BMI	23.78 ± 3.18	23.04 ± 2.89	24.67 ± 2.36	2.4 × 10^−6^	0.087	3.4 × 10^−4^
WHR	0.81 ± 0.06	0.81 ± 0.06	0.82 ± 0.06	0.284	0.517	0.238
SBP (mm Hg)	113.24 ± 12.20	112.12 ± 11.39	118.53 ± 13.65	2.4 × 10^−5^	0.800	1.0 × 10^−4^
DBP (mm Hg)	75.25 ± 8.79	73.72 ± 7.82	76.64 ± 8.15	0.003	0.149	0.014
GLU (mmol/L)	5.38 ± 0.73	5.33 ± 0.71	5.31 ± 0.56	0.537	0.272	0.607
TC (mmol/L)	4.71 ± 0.88	4.74 ± 0.83	4.97 ± 0.86	0.031	0.243	0.009
TG (mmol/L)	1.26 ± 0.96	1.14 ± 0.65	1.32 ± 1.31	0.088	0.224	0.349
HDL-C (mmol/L)	1.64 ± 0.33	1.63 ± 0.31	1.63 ± 0.32	0.868	0.598	0.816
LDL-C (mmol/L)	2.54 ± 0.69	2.58 ± 0.69	2.75 ± 0.72	0.031	0.131	0.012

BMI: body mass index, WHR: waist-to-hip ratio, SBP: systolic blood pressure, DBP: diastolic blood pressure, GLU: plasma glucose, TC: total cholesterol, TG: triglyceride, HDLC: high-density lipoprotein cholesterol, LDLC: low-density lipoprotein cholesterol.

^a^Dominant model: GA+AA vs GG.

^b^Recessive model: AA vs GG+AG.

For SNP rs17209251, the GG genotype was associated with significantly higher SBP in the additive and recessive models (*P* < 0.0004 for both comparisons; Table 5). In the recessive model, the GG genotype was associated with a significantly lower triglycerides concentration as compared with A allele carriers (*P* < 0.0004).

**Table 5.** Associations of cardiovascular risk factors with rs17209251 genotypes/alleles

Variable	AA (*n* = 543)	AG (*n* = 192)	GG (*n* = 27)	*P* (Additive model)	*P* (Dominant model^a^)	*P* (Recessive model^b^)
Age (years)	41.66 ± 10.39	41.60 ± 11.65	46.33 ± 6.81			
BMI	23.56 ± 3.00	23.39 ± 3.06	24.42 ± 2.45	0.243	0.862	0.123
WHR	0.81 ± 0.06	0.82 ± 0.06	0.80 ± 0.03	0.264	0.317	0.094
SBP (mm Hg)	112.76 ± 11.69	113.68 ± 12.85	123.78 ± 12.39	2.2 × 10^−5^	0.035	5.5 × 10^−6^
DBP (mm Hg)	74.25 ± 8.31	75.33 ± 8.23	79.22 ± 7.45	0.005	0.019	0.003
GLU (mmol/L)	5.37 ± 0.76	5.30 ± 0.52	5.15 ± 0.42	0.179	0.072	0.144
TC (mmol/L)	4.72 ± 0.87	4.90 ± 0.84	4.54 ± 0.60	0.015	0.042	0.064
TG (mmol/L)	1.25 ± 0.93	1.13 ± 0.80	0.96 ± 0.23	0.097	0.034	2.1 × 10^−5^
HDL-C (mmol/L)	1.62 ± 0.33	1.67 ± 0.31	1.59 ± 0.25	0.165	0.150	0.458
LDL-C (mmol/L)	2.55 ± 0.71	2.71 ± 0.67	2.39 ± 0.54	0.007	0.029	0.131

BMI: body mass index, WHR: waist-to-hip ratio, SBP: systolic blood pressure, DBP: diastolic blood pressure, GLU: plasma glucose, TC: total cholesterol, TG: triglyceride, HDLC: high-density lipoprotein cholesterol, LDLC: low-density lipoprotein cholesterol.

^a^Dominant model: AG+GG vs AA.

^b^Recessive model: GG vs AA+AG.

For SNP rs2963156, no genotype/allele was significantly associated with the evaluated cardiovascular risk factors in the additive or dominant models (*P* > 0.0004; Table 6). However, in the recessive model, the TT genotype was associated with significantly lower plasma glucose, triglycerides, and LDL cholesterol as compared with C allele carriers (*P* < 0.0004).

**Table 6.** Associations of cardiovascular risk factors with rs2963156 genotypes/alleles

Variable	CC (*n* = 590)	CT (*n* = 151)	TT (*n* = 21)	*P* (Additive model)	*P* (Dominant model^a^)	*P* (Recessive model^b^)
Age (years)	41.96 ± 10.48	41.13 ± 11.86	42.67 ± 4.19			
BMI	23.54 ± 3.01	23.33 ± 2.92	27.75 ± 2.95	0.162	0.830	0.080
WHR	0.81 ± 0.06	0.82 ± 0.06	0.79 ± 0.02	0.100	0.358	0.001
SBP (mm Hg)	112.93 ± 11.60	114.51 ± 14.33	118.33 ± 10.98	0.080	0.095	0.081
DBP (mm Hg)	74.44 ± 8.26	75.53 ± 8.75	75.83 ± 6.24	0.306	0.125	0.554
GLU (mmol/L)	5.38 ± 0.75	5.25 ± 0.48	4.93 ± 0.19	0.005	4.8 × 10^−4^	2.1 × 10^−7^
TC (mmol/L)	4.75 ± 0.86	4.82 ± 0.90	4.43 ± 0.50	0.182	0.695	0.014
TG (mmol/L)	1.23 ± 0.90	1.14 ± 0.85	0.91 ± 0.26	0.213	0.139	2.0 × 10^−4^
HDL-C (mmol/L)	1.64 ± 0.33	1.62 ± 0.30	1.69 ± 0.22	0.653	0.643	0.363
LDL-C (mmol/L)	2.58 ± 0.69	2.68 ± 0.73	2.14 ± 0.20	0.006	0.490	2.0 × 10^−7^

BMI: body mass index, WHR: waist-to-hip ratio, SBP: systolic blood pressure, DBP: diastolic blood pressure, GLU: plasma glucose, TC: total cholesterol, TG: triglyceride, HDLC: high-density lipoprotein cholesterol, LDLC: low-density lipoprotein cholesterol.

^a^Dominant model: CT+TT vs CC.

^b^Recessive model: TT vs CC+CT.

## DISCUSSION

Using a multimarker approach and quantitative analysis, we examined whether variants in the GR gene were associated with cardiovascular risk factors among the Chinese Han population. Of the 19 makers studied, associations with 5 markers were evident; associations with the other 14 makers were nonsignificant. Although the N363S, *Bcl* I, ER22/23EK, and A3669G polymorphisms of the GR gene have been identified among white and African-American populations,^23^^–^^25^ only the *Bcl* I polymorphism was found in our study. The frequency of the minor allele of the *Bcl* I polymorphism in the present study was similar to that among other Asian populations and among Western populations.^26^^–^^28^ Duan et al did not detect the N363S, ER22/23EK, or A3669G polymorphism among another Chinese Han population or among a Japanese population.^27^ The D401H polymorphism, which was identified in a 43-year-old Colombian woman, was not detected in the present study.

The *Bcl* I (rs41423247) polymorphism is a C→G nucleotide change downstream of the exon 2–intron 2 junction of the GR gene.^6^ Our study showed that homozygous (GG) carriers had a higher BMI and SBP, and lower plasma glucose and triglycerides, as compared with C allele carriers (*P* < 0.0004). This finding agreed with the results of previous studies, which found that *Bcl* I polymorphisms were associated with higher blood pressure and/or higher prevalence of hypertension.^29^ *Bcl* I polymorphisms were found to be associated with variation in sensitivity to exogenous glucocorticoids in rheumatoid arthritis and healthy individuals.^30^^,^^31^ In another study, the GG genotype was more frequent in a group with personal and parental hypertension.^29^ As previously suggested,^32^ this phenomenon may be due to a possible linkage disequilibrium between the *Bcl* I polymorphism and other polymorphisms located at regulatory regions that might influence the observed phenotype. In obese persons, homozygous G allele carriers had higher levels of insulin and blood glucose and were more insulin-resistant than noncarriers of the G allele.^16^ In addition, Weaver et al showed that unrelated, nondiabetic, premenopausal white women with the GG genotype had a higher fasting insulin level than did normal-weight controls. We found that plasma glucose was lower among homozygous GG carriers than among C allele carriers. However, data on fasting insulin were not available in our study. Van Rossum reported that among middle-aged adults, the G allele of the *Bcl* I polymorphism was associated with increased abdominal obesity.^6^ In our study, only GG homozygotes had higher BMI values. In summary, data on the *Bcl* I polymorphism are conflicting with respect to its association with body composition and metabolic parameters.^24^^,^^33^ A possible explanation for these contradictory findings is that hypersensitivity to GCs due to *Bcl* I polymorphism has consequences that differ throughout life.

For SNP rs10052957 (Tth111I), A allele carriers had significantly lower SBP, total cholesterol, and LDL-C than did GG carriers (*P* > 0.05). Although the Tth111I polymorphism is not functional by itself, it might be functionally relevant in combination with ER22/23EK. The ER22/23EK polymorphism is associated with relative GC resistance and a healthier metabolic profile, ie, lower cholesterol levels and increased insulin sensitivity.^20^^,^^34^ It is not known if the Tth111I variant of the GR gene is essential in the associations of the ER22/23EK polymorphism or if its presence at the same allele is coincidental and does not influence the effects of the ER22/23EK variant. However, in our sample we found no variation in ER22/23EK (or in N363S, A3669G, or D401H).

The rs7701443 AA genotype is associated with higher BMI and SBP (*P* < 0.0004), although no significant association was found in the dominant model. The results suggest that the rs7701443 AA genotype is associated with increased sensitivity to GC. Krupovesa et al reported that the rs7701443 G allele was associated with corticosteroid resistance in children with Crohn disease.^35^ Lower plasma glucose, triglyceride, and LDL cholesterol were observed among rs2963156 TT carriers (*P* < 0.0004). Krupovesa et al reported that rs2963156 was associated with corticosteroid dependence in a recessive model.^35^ For rs17209251, which is in high LD with rs17209258 and rs4986593, the G allele was associated with higher SBP but lower triglycerides, as compared with A allele carriers (*P* < 0.0004).

Our findings suggest that variation in the GR gene has a role in susceptibility to cardiovascular disease. Recently, a few large-scale genome-wide association studies of East Asians have noted associations with BMI and blood pressure.^36^^,^^37^ Associations with the GR gene in the present study will assist in detecting loci that were missed in the large-scale studies. However, these associations must be investigated further in a large case-control study and in cohorts. The statistical power (α = 0.0004) for each SNP/trait in our study was not sufficient (rs41423247: 0.24–0.80; rs10052957: 0.79–0.99; rs7701443: 0.73–0.99; rs17209251: 0.20–0.85, rs2963156: 0.06–0.99), because of the small sample size. We therefore cannot exclude the possibility that we failed to find associations between the GR gene and other traits, due to type II error. Furthermore, future functional studies should assess the implications of the observed associations.
